# p53 activation via pharmacologic MDM2 inhibition constrains exhaustion and enhances antitumor function of human CD8+ T cells

**DOI:** 10.3389/fimmu.2026.1910235

**Published:** 2026-10-16

**Authors:** Alexandra Boix de Jesus, Hy B. Dao, Anthony Imani, Gabriel A. Rodriguez, Michael S. Shehata, Rachana R. Chandran, Stuart A. Fine, Sin Y. Lim, Michelle To, Serena Y. Lofftus, Nicolas Pedroncelli, Fritz C. Eilber, Brigitte N. Gomperts, Tyler R. McCaw, Cristina Puig-Saus, Joseph G. Crompton

**Affiliations:** 1 Department of Surgery, UCLA, Los Angeles, CA, United States; 2 Division of Surgical Oncology, UCLA, Los Angeles, CA, United States; 3 Division of Hematology/Oncology, UCLA, Los Angeles, CA, United States; 4 Department of Pediatrics, UCLA, Los Angeles, CA, United States; 5 David Geffen School of Medicine, UCLA, Los Angeles, CA, United States; 6 UCLA Bioengineering, UCLA, Los Angeles, CA, United States; 7 Jonsson Comprehensive Cancer Center, Sarcoma Oncology Program, UCLA, Los Angeles, CA, United States

**Keywords:** CD8, exhaustion, immunotherapy, metabolic fitness, p53, T-cell memory

## Abstract

Adoptive T-cell therapies using chimeric antigen receptors (CARs) or engineered T-cell receptors (TCRs) have transformed cancer treatment but remain limited by poor persistence and progressive T-cell exhaustion. Manufacturing strategies that promote memory T-cell formation may improve therapeutic durability. Here we investigated whether transient pharmacologic activation of the p53 pathway influences the differentiation and function of human CD8+ T cells. p53 expression was minimal in quiescent naïve CD8+ T cells but was rapidly induced following T-cell receptor activation. Pharmacologic inhibition of the p53 negative regulator MDM2 increased p53 protein levels and promoted expansion of central memory and stem-like memory T-cell subsets during clonal proliferation. Treated cells displayed enhanced mitochondrial fitness, increased polyfunctional cytokine production, and transcriptional programs consistent with memory differentiation and reduced exhaustion. Importantly, TCR-engineered CD8+ T cells generated in the presence of the MDM2 inhibitor Navtemadlin demonstrated superior tumor-cell killing *in vitro* compared with conventionally manufactured cells. These findings identify transient p53 activation as a pharmacologic strategy to enhance memory programming during T-cell manufacturing and suggest a clinically feasible approach to improve the efficacy of adoptive T-cell immunotherapies.

## Highlights

- Transient pharmacologic activation of p53 promotes memory differentiation and constrains exhaustion in human CD8+ T cells.
- MDM2 inhibition expands central memory T-cell subsets during clonal proliferation.
- p53 activation enhances metabolic fitness and polyfunctional cytokine production.
- TCR-engineered T cells generated with p53 activation demonstrate improved tumor killing.
- Pharmacologic p53 modulation represents a practical strategy to improve engineered T-cell manufacturing.

• Take-home message: Transient pharmacologic activation of p53 during T-cell manufacturing enhances memory programming and constrains exhaustion to improve antitumor function.

## Introduction

Adoptive T-cell therapies using chimeric antigen receptors (CARs) or genetically engineered T-cell receptors (TCRs) have emerged as powerful treatments for hematologic malignancies and are increasingly being explored for solid tumors (1). Despite these advances, therapeutic responses remain inconsistent and many patients ultimately relapse. A major limitation of current approaches is the poor persistence and progressive exhaustion of transferred T cells following infusion. Increasing evidence indicates that the differentiation state of T cells at the time of infusion strongly influences therapeutic efficacy. Less differentiated subsets, including stem-like memory (Tscm) and central memory (Tcm) T cells, display superior proliferative potential, persistence, and antitumor activity compared with terminally differentiated effector populations (2–4).

Conventional manufacturing protocols for engineered T-cell therapies often drive rapid proliferation and differentiation, leading to a predominance of effector-like cells that exhibit limited longevity. Therefore, strategies that promote memory formation while preserving expansion capacity are of considerable interest (5–7). Understanding the molecular pathways that regulate T-cell fate decisions during early activation may reveal opportunities to optimize manufacturing protocols.

The tumor suppressor protein p53 is a central regulator of cellular stress responses and plays critical roles in cell-cycle arrest, apoptosis, DNA repair, and metabolic regulation (8, 9). Although the p53 pathway has been extensively studied in cancer biology, its function in human T-cell differentiation remains incompletely understood. p53 activity is tightly controlled by the E3 ubiquitin ligase MDM2, which targets p53 for proteasomal degradation through a negative feedback loop (10–12). Pharmacologic inhibitors of the MDM2–p53 interaction have been developed to activate p53 signaling and are currently under clinical investigation for cancer treatment (13–18).

Given the central role of p53 in cellular homeostasis and stem-cell regulation, we hypothesized that transient activation of the p53 pathway might influence the differentiation trajectory of activated CD8+ T cells. In this study, we examined the effects of pharmacologic MDM2 inhibition on human CD8+ T cells during early activation and clonal expansion. Our results demonstrate that transient p53 activation promotes the formation of memory-like T cells, enhances metabolic fitness, and improves the antitumor activity of engineered TCR-T cells. These findings suggest a pharmacologic strategy to enhance the quality and therapeutic potential of adoptively transferred T cells.

## Materials and methods

### Human T-cell isolation and activation

Human peripheral blood mononuclear cells (hPBMCs) were pooled from multiple healthy, de-identified donors from UCLA CDU CFAR Centralized Support Core. Cells were cryopreserved in a CryoStor (10% v/v; Stemcell Technologies) and stored at −80 °C until use. Prior to experimentation, hPBMCs were thawed, washed with Gibco™ PBS and then cultured at 37 °C in a humidified incubator using Gibco™ RPMI 1640 medium (ATCC modification, ThermoFisher Scientific) supplemented with 10% v/v fetal bovine serum (FBS; Gibco) and 1% v/v penicillin-streptomycin (Gibco). Cells were stimulated on a coated plate with CD3 monoclonal antibody (OKT3, Functional Grade; eBioscience™, ThermoFisher Scientific) and CD28 monoclonal antibody (CD28.2; ThermoFisher Scientific) at a 2/5 ratio respectively in addition to recombinant human IL-2 (120 IU) (ThermoFisher Scientific). Naïve CD8+ T cells were isolated by magnetic bead-based negative selection using the EasySep™ Human Naïve CD8+ T Cell Isolation Kit, STEMCELL Technologies EasySep™.

### Collection of tumor-infiltrating lymphocytes from sarcoma specimens

Tumor specimens from two sarcoma patients were cut into small fragments of 1-2mm^3^ and placed individually in a suspension 24-well plate with 2mL of media containing Gibco™ RPMI 1640 medium (ATCC modification, ThermoFisher Scientific) supplemented with 10% v/v human AB serum (Gemini Bio) and 1% v/v penicillin-streptomycin (Gibco) plus 6000IU/mL of human IL-2. Collected TILs were then expanded for another period of 14 days prior to cryopreservation in Cryostor (CS10) as previously described (19). Thawed cells were used for downstream analysis in this study.

### Pharmacologic activation of p53

To activate the p53 pathway, cells were treated with small-molecule inhibitors of the MDM2–p53 interaction. Multiple compounds were initially evaluated, including Nutlin-3a, Alrizomadlin, SAR405838, and Navtemadlin obtained from MedChemExpress. Based on potency and cellular responses, Navtemadlin was selected for subsequent experiments. Cells were exposed to graded concentrations of inhibitors at the beginning of cell culture. Cells in the control (or untreated) group were exposed to the same final concentration of DMSO (2uL per 10mL of media) as the drug-treated group to control for vehicle effects. Experiments were performed with three biological replicates unless otherwise indicated.

### Flow cytometry staining, acquisition, and data analysis

T-cell phenotype and intracellular protein expression were analyzed by multiparameter flow cytometry. Memory subsets were defined using canonical markers distinguishing naïve, central memory, effector memory, and stem-like memory populations. Intracellular staining was performed to quantify p53 expression as well as functional molecules including interferon-γ and granzyme B. Flow cytometry and FACS staining were performed using fluorochrome-conjugated antibodies specific to the following targets: CD3 (clone UCHT1, Biolegend), CD8 (clone RPA-T8, Biolegend), CD45RO (clone UCHL1, Biolegend), CCR7 (clone G043H7, Biolegend), CD62L (clone DREG-56, Biolegend), CD95 (clone DX2, Biolegend), IFN-γ (clone B27, Biolegend), Granzyme B (GzB; clone GB11, Biolegend), p53 (clone DO-7, Biolegend), and the THETM NWSHPQFEK Tag Antibody FITC (GenScript). Live/dead discrimination was performed using the LIVE/DEAD Fixable Near-IR Dead Cell Stain Kit (ThermoFisher Scientific) with excitation at 633 or 635 nm. For all assessments of intracellular cytokine production, specifically IFN-γ and GzB, cells were incubated for 5 hours with Invitrogen™ eBioscience™ Cell Stimulation Cocktail (plus protein transport inhibitors) diluted to 1X from 500X stock, before fixing with IC Fixation Buffer and permeabilizing with Permeabilization Buffer diluted to 1X from 10X (both from Invitrogen), according to the manufacturer’s instructions. For intracellular p53 detection, cells were fixed using True-Nuclear Fix Concentrate 4X diluted in True-Nuclear Fix Diluent and permeabilized with True-Nuclear 10X Permeabilization Buffer, all from the True-Nuclear Transcription Factor Buffer Set (Biolegend). Flow cytometry data were acquired on an Attune™ NxT Flow Cytometer (ThermoFisher Scientific), and FACS sort was performed on a SORP FACSAria™ II High-Speed Cell Sorter (BD Biosciences). All flow cytometry and cell sorting procedures were performed at the UCLA Flow Cytometry Core Facility. The flow data were analyzed using FlowJo v10.10.0 Software. Fluorescence-minus-one (FMO)and unstained controls were included to assist in the gating strategy.

### Quantitative PCR analysis of p53 isoforms

RNA was isolated from sorted T-cell subsets and converted to cDNA. Quantitative PCR was performed to evaluate expression of full-length p53 and major p53 isoforms. Relative expression levels were calculated using standard normalization methods. Naïve CD8+ T cells were isolated from PBMCs, separated into two groups (untreated and 2 µM NAV-treated groups), and split into triplicate. After 5 days, RNA extraction was performed by the TCGB core. Briefly, RNA concentration and purity were confirmed quantitatively using Nanodrop spectrophotometry, while RNA integrity was assessed qualitatively using the Agilent Tape Station. RNA-Seq libraries were prepared using the KAPA Stranded mRNA-Seq Kit. The workflow consists of mRNA enrichment and fragmentation, first strand cDNA synthesis using random priming followed by second-strand synthesis, converting cDNA: RNA hybrid to double-stranded cDNA (dscDNA), and incorporating dUTP into the second cDNA strand. cDNA generation was followed by end repair to generate blunt ends, A-tailing, adaptor ligation, and PCR amplification. Different adaptors were used to multiplex the samples in one lane. Sequencing was performed on an Illumina Novaseq X Plus for PE 2x50 run. A data quality check was performed using Illumina SAV. Demultiplexing was performed using the Illumina Bcl2fastq (v2.19.1.403) software.

### Transcriptomic analysis

Bulk RNA sequencing was performed on activated CD8+ T cells cultured with or without Navtemadlin. Differential gene expression analysis and gene set enrichment analysis were conducted to evaluate transcriptional programs associated with memory differentiation, effector function, and exhaustion. Alignment was performed using STAR with the human reference genome, GRCh38. The Ensembl transcript release GRCh38.107 GTF was used for annotation of gene features. Raw gene-level count data were filtered to exclude lowly expressed genes (retaining genes with more than 10 total counts) and those lacking a defined name. After raw read counts were rounded to the nearest integer, DESeq2 (v1.48.1) was used to perform differential gene expression analysis, where raw counts were normalized by dividing by sample-specific size factors, which were estimated using the median-of-ratios method. Differential expressions were assessed using DESeq2’s built-in Wald test by applying the Benjamini-Hochberg correction for multiple testing. Genes with an absolute log2 fold change > 1 and adjusted p-value < 0.01 were considered significantly differentially expressed. For gene set enrichment analysis (GSEA), an FDR<0.05 was deemed significant.

Principal component analysis (PCA) plots were generated using the plotly package (v4.11.0) to illustrate the global expression patterns and sample clustering. Heatmaps of normalized expression counts were constructed using pheatmap (v1.0.13) to demonstrate unsupervised hierarchal clustering of untreated and NAV-treated samples. GSEA was performed using fgsea (v1.34.0), and enrichment plots were created using gseaplot2 from clusterProfiler (v4.16.0). Volcano plots were generated using the EnhancedVolcano R package (v1.26.0). Bar plots highlighting genes of interest were generated using ggplot2 (v3.5.2).

To visualize Gene Ontology (GO) enrichment results, the top 350 GO terms (ranked by adjusted p-value) were selected from clusterProfiler’s enrichGO (R) enrichment analysis. These terms and their associated p-values were uploaded to REVIGO to reduce redundancy by clustering semantically similar GO terms. REVIGO was run with default settings (similarity cutoff = 0.7, semantic similarity = SimRel, species database = Homo sapiens). The resulting tree map file was inputted into Circular Gene Ontology (CirGO) software (v2.0) to generate circular diagrams representing the enriched GO categories organized into broader biological processes through hierarchical clustering, with the top 100 categories visualized.

### Metabolic analysis

Mitochondrial function was assessed using a mitochondrial stress assay that measures oxygen consumption rate and spare respiratory capacity following sequential injection of metabolic inhibitors. The oxygen consumption rate (OCR) was measured using a Seahorse XFe96 Extracellular Flux Analyzer (Agilent Technologies). A total of 450,000 cells/well were seeded in a poly-D-lysine (PDL)-coated Seahorse XF96 microplate. The cell plate was centrifuged at 300 x g for 5 min without breaking so that cells could adhere to the Seahorse plate. The cells were washed twice with Seahorse DMEM assay medium supplemented with 5 mM HEPES, 5 mM glucose, 2 mM glutamine, and 1 mM sodium pyruvate; pH 7.4. Compounds were injected sequentially during the assay resulting in final concentrations of 2 μM oligomycin, 1.5 μM FCCP, and 1 μM rotenone and 2 μM antimycin A. Respirometry parameters and ATP production rates were calculated as described previously (20, 21).

### Generation of TCR-engineered T cells

Naïve CD8+ T cells were stimulated and transduced with tumor-specific TCR constructs targeting tumor antigen MAGE-A4. Transduction was performed during early activation, and cells were expanded for functional evaluation. To test the effect of 1 µM Navtemadlin on CD8+ T-cell effector function, CD8+ T cells were genetically engineered to express MAGE-A4 TCR and tumor cell killing was measured using the Incucyte S3 Live Cell Analysis instrument. Two days after naïve CD8+ T cell isolation and activation with CD3:CD28 antibody ratio of 2:5 and IL-2; 120IU, retronectin coated plates were used to spinoculate MAGE-A4 virus for 2h at 2000G and CD8+ T cells were added without changing the media to maintain the remaining Navtemadlin concentration added on the first day (1 µM) and a final spin of 10’ at 2000G was performed. Four days post-transduction, before co-culture, CD8+ T cells were checked for MAGE-A4 expression to assess TCR transduction with the THETM NWSHPQFEK Tag Antibody FITC (GenScript). On day 6, CD8+ T cells were rinsed and transferred to a fresh media containing no IL-2 to begin the co-culture with MAGE-A4 positive melanoma cells (M407) which was genetically modified to express nuclear red fluorescence protein (RFP). Tumor cell death was measured using the Incucyte S3 Live Cell Analysis instrument for 160 hours by taking pictures every 2 hours.

### Tumor killing assays

Engineered T cells were co-cultured with antigen-expressing tumor cell lines at multiple effector-to-target ratios. Cytotoxicity was assessed using real-time imaging and quantification of tumor cell clearance over time. CD8+ T cells were co-cultured with MAGE-A4 positive melanoma cells (M407) genetically modified to express nuclear red fluorescence protein (RFP). Tumor cell death was measured using the Incucyte S3 Live Cell Analysis instrument for 160 hours by taking pictures every 2 hours.

## Results

### p53 is induced during early T-cell activation

Naïve CD8^+^ T cells are maintained in a quiescent state under homeostatic conditions (22, 23). Upon activation through the T-cell receptor (TCR), these cells exit quiescence and undergo rapid clonal expansion and differentiation into effector and memory subsets. Because p53 regulates cellular quiescence in several cell types, including hematopoietic stem cells (24), we first investigated whether p53 contributes to maintaining the quiescent state of naïve CD8^+^ T cells.

Naïve CD8^+^ T cells exhibited minimal basal expression of p53 protein (**Figure 1A**). However, following TCR stimulation, p53 expression increased progressively during early activation, indicating that p53 induction occurs after T-cell activation rather than maintaining quiescence in resting cells.

**Figure 1:**
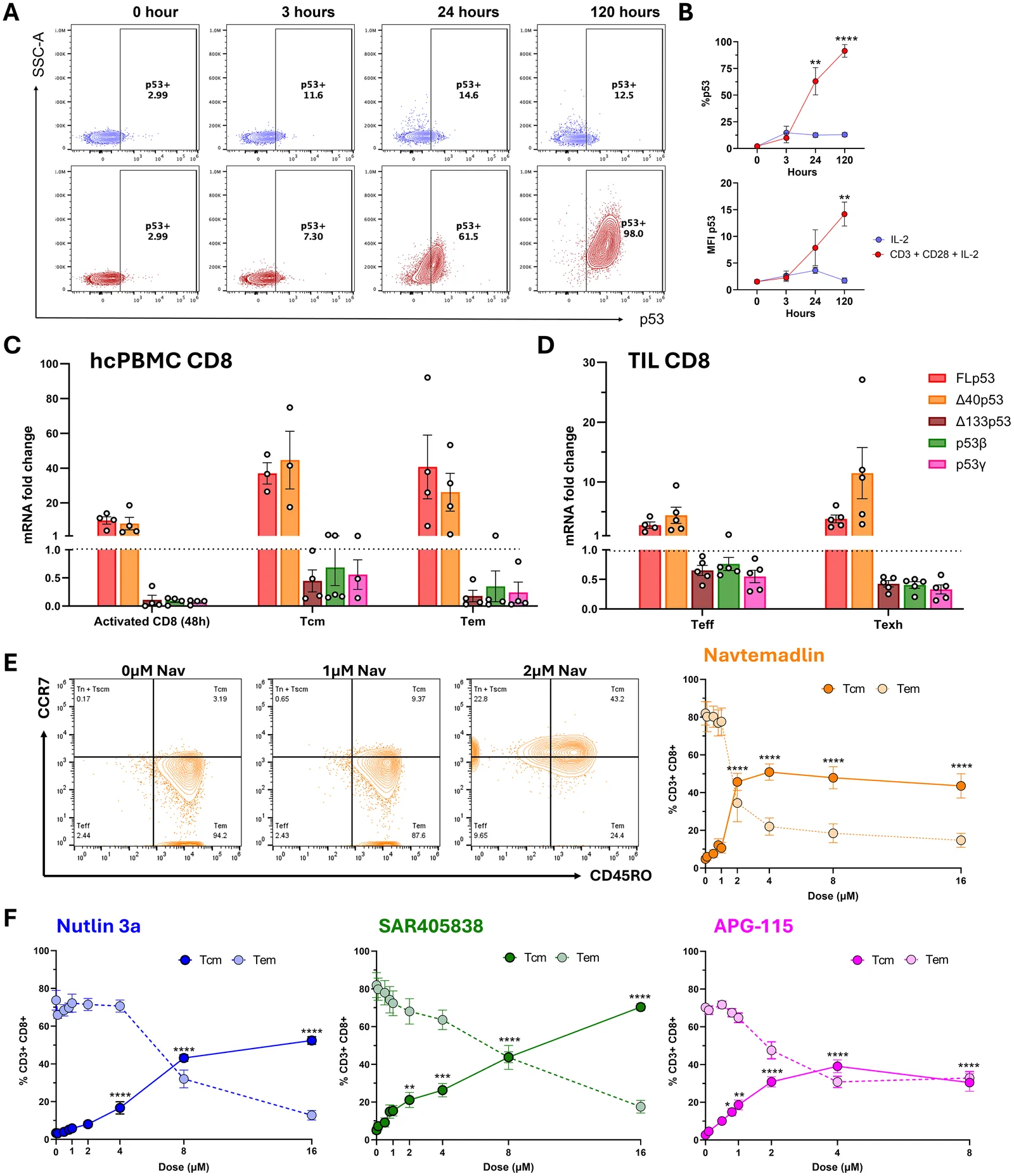
p53 is induced during early T-cell activation and MDM2 inhibition promotes expansion of memory T cells. Isolated naïve CD8+ T cells were cultured in IL-2 with (red) or without (blue) anti-CD3 and anti-CD28 antibodies **(A)**. Representative flow plot of p53 expression was assessed at different timepoints as shown. **(B)** Quantification of p53 expression and MFI were shown (n ≥ 3 biological replicates). **(C)** Fold change of p53 isoforms’ mRNAs isolated from activated or sorted CD8+ T-cell subsets compared to baseline naïve CD8 T cells. Naïve CD8+ T cells were activated with CD3/CD28/IL-2 for 120 hours and sorted for central memory (Tcm, CD45RO+ CD62L+) and effector memory (Tem, CD45RO+ CD62L-) prior to mRNA extraction (n ≥ 3 biological replicates). **(D)** Thawed TILs from 2 different sarcoma patients with technical replicates were sorted for effector (Teff, PD1+ Tim3-) and exhausted (Texh, PD1+ Tim3+) CD8+ T cells and assessed for p53 mRNA expression compared to naïve. **(E)** Flow plots of naïve CD8+ T cells gated on live CD45+ CD3+ CD8+ when activated with CD3/CD28/IL-2 for 120 hours in the presence of Navtemadlin (left). Frequency of Tcm and Tem were quantified (n ≥ 3 biological replicates) as shown (right). **(F)** MDM2 inhibition with Alrizomadlin (APG-115), SAR405838, or Nutlin-3a in technical replicates shows similar results. Asterisks indicate statistical significance determined by one-way ANOVA followed by Dunnett’s multiple comparisons test: *p < 0.05, **p < 0.01, ***p < 0.001, ****p < 0.0001. Bars represent mean, and error bars indicate SEM.

Previous studies have shown that p53 can suppress IL-2–dependent proliferation of murine CD4^+^ T cells in the absence of cognate antigen stimulation (25). We therefore asked whether IL-2 alone was sufficient to induce p53 in human CD8^+^ T cells. In contrast to TCR stimulation, culture in IL-2 alone resulted in only modest p53 induction, indicating that robust p53 expression requires TCR signaling (**Figure 1B**).

We next examined whether p53 isoforms were expressed at a transcriptional level following activation. The TP53 gene encodes multiple isoforms generated through alternative splicing, promoter usage, and translation initiation (26–28). These isoforms can modulate the activity of full-length p53 and influence diverse cellular processes including apoptosis, DNA repair, senescence, and stem cell maintenance (29–32). Quantitative analysis revealed substantial upregulation of full-length p53 transcripts and selected isoforms following 48 hours of activation from naïve, with particularly high expression in memory CD8+ T-cell subsets found on day 5 post initial activation (**Figure 1C**). Compared to these memory subsets, full-length p53 transcripts and its isoforms were reduced in highly differentiated or exhausted CD8+ T-cell populations isolated from patients’ tumor infiltrating lymphocytes (TILs) (**Figure 1D**). This observation was further supported by flow cytometry analysis when naïve CD8+ T cells were isolated and activated in culture for 5 days to measure p53 expression and its mean fluorescence intensity (MFI) within each subset of CD8+ T cells (**Supplementary Figures S1A, B**).

Together, these findings demonstrate that p53 expression is induced during early T-cell activation and that p53 isoforms are enriched in memory CD8^+^ T-cell populations, suggesting a potential role for the p53 pathway in regulating T-cell differentiation.

### MDM2 inhibition promotes expansion of memory T-cell subsets

Having observed that p53 isoforms are enriched in memory CD8+ T-cell populations, we next investigated whether pharmacologic activation of the p53 pathway could influence T-cell differentiation following activation from a naïve state. To test this, we treated human naïve CD8+ T cells with Navtemadlin, a small-molecule inhibitor of the p53 negative regulator MDM2, concurrently with or without TCR stimulation. Inhibition of MDM2 increased p53 protein levels both activated and resting CD8+ T cells (**Supplementary Figure S1C**), confirming effective pharmacologic activation of the pathway. We then next examined how MDM2 affected CD8+ T-cell subset composition following TCR stimulation. Compared to other MDM2 inhibitors tested, Navtemadlin produced the most pronounced effects on T-cell differentiation at lower doses (**Figures 1E, F**). Treatment with Navtemadlin significantly increased the frequency of central memory T cells (Tcm), while concomitantly reducing the proportion of effector memory T cells (Tem) within the activated CD8+ T-cell population. These results suggest that pharmacologic activation of p53 through MDM2 inhibition promotes differentiation towards a memory T-cell phenotype during early CD8+ T-cell activation.

### Transcriptional profiling reveals a shift toward memory programs and constrained exhaustion

To further characterize whether pharmacologic inhibition of MDM2 increases p53 expression and promotes the expansion of memory CD8+ T-cell subsets, we next investigated the impact of this pathway at the transcriptional level. Bulk RNA sequencing was performed on activated CD8+ T cells from 3 different donors in the presence or absence of the MDM2 inhibitor Navtemadlin (**Figure 2A** left).

**Figure 2:**
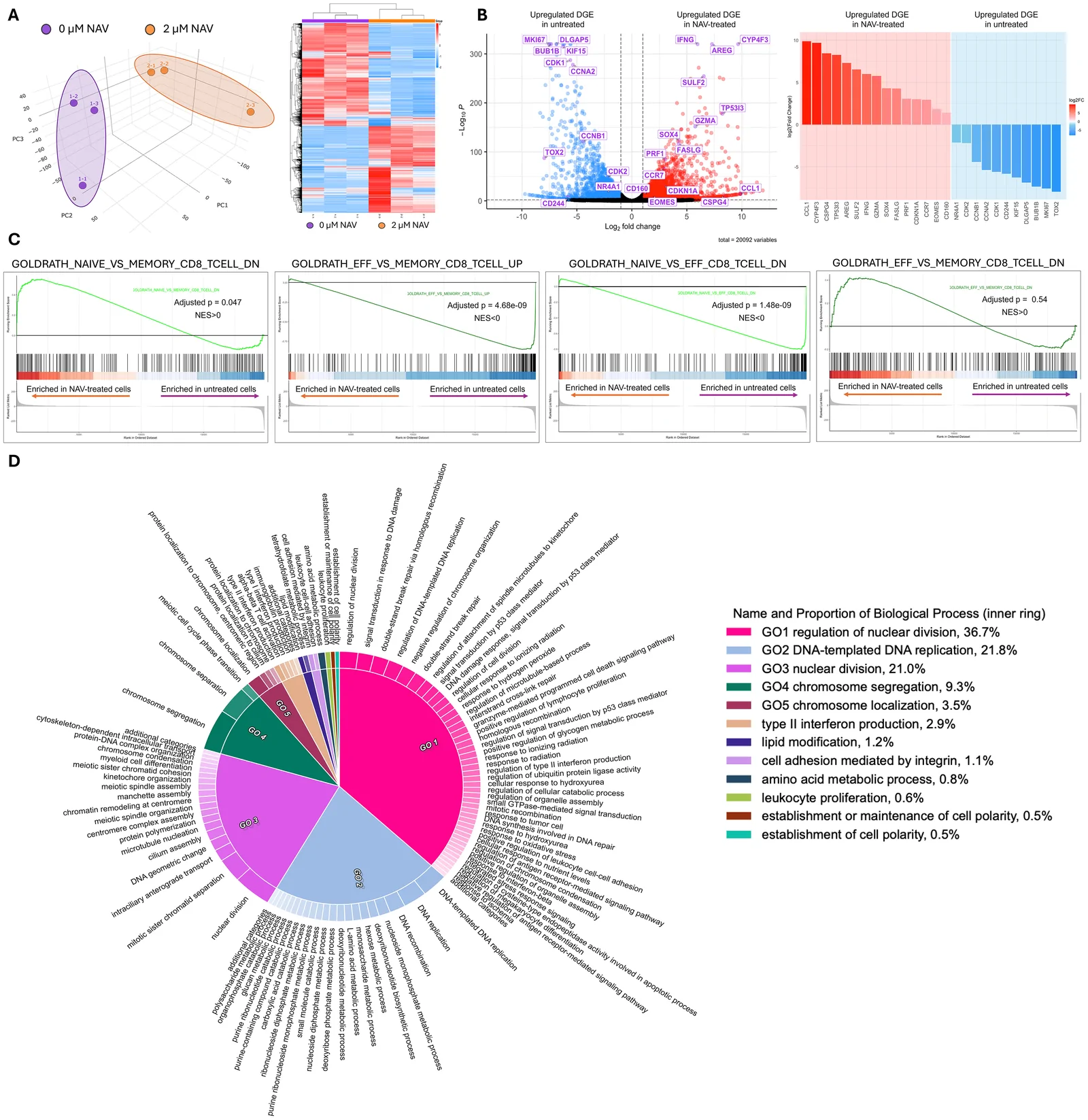
Gene expression profile shows reduced exhaustion and increased memory genes in CD8 T cells treated with MDM2 inhibitor. **(A)** Principal component analysis (PCA) of bulk RNA-seq between untreated (purple) and NAVtreated (orange) CD8+ T-cell samples when grouped based on overall transcriptomic similarity (n = 3 biological replicates). Hierarchical clustering heatmap of 6,313 differentially expressed genes (|log₂FoldChange| > 1, padj < 0.01). Rows represent genes and columns represent biological replicates. Red and blue indicate relative upregulation and downregulation (z-score scaled expression), respectively. **(B)** Volcano plot shows differential gene expression (DGE) between untreated and NAV-treated groups. Each dot represents a significantly upregulated gene in the group as shown (|log₂FoldChange| > 1, padj < 0.01). Key memoryassociated and effector-associated genes (purple) are labeled. Bar plot of log₂ fold change shows top upregulated genes within each group. **(C)** GSEA shows NAV-treated cells have significant positive enrichment of genes upregulated in memory and significant negative enrichment of genes upregulated in effector. Normalized enrichment score (NES), running enrichment score (green line), and ranked genes (barcode) are shown. Genes ranked closest to the left (rank = 1) are most upregulated in NAV-treated cells, while those furthest to the right are most upregulated in untreated cells. Adjusted p value of less than 0.05 was deemed significant. **(D)** CirGO visualization of enriched Gene Ontology (GO) biological process terms for differentially expressed genes. Inner rings display higher order GO categories, and outer rings show individual terms.

Transcriptomic analysis revealed a clear separation between treated and untreated cells, indicating that MDM2 inhibition induces a distinct transcriptional state in activated CD8+ T cells (**Figure 2A** right). Navtemadlin-treated cells exhibited enrichment of gene expression programs associated with memory T-cell differentiation such as CCL1, CCR7, and EOMES (33), whereas untreated cells displayed stronger transcriptional signatures linked to effector differentiation and T-cell exhaustion such as TOX, NR4A1, CD244, and CCNA2 (**Figure 2B**) (34). Gene set enrichment analysis further confirmed significant enrichment of memory-associated pathways following p53 activation. For example, the Goldrath_Naive_vs_Memory_CD8_TCELL_DN gene set represents genes that are down in naïve relative to memory, or in other words, genes that are relatively high in memory T cells. These genes were significantly enriched in the Navtemadin treatment as shown (**Figure 2C**). In contrast, gene sets associated with effector differentiation were preferentially enriched in untreated cells. In the Goldrath_Eff_vs_Memory_CD8_TCELL_UP, genes that are upregulated in the effector T cells were significantly enriched in the untreated cells.

Gene ontology analysis revealed enrichment of differentially expressed genes involved in processes such as nuclear division and DNA-templated DNA replication, consistent with transcriptional programs regulating T-cell proliferation and differentiation (**Figure 2D**). Together, these findings indicate that pharmacologic activation of the p53 pathway promotes a transcriptional program favoring memory CD8+ T-cell differentiation.

### MDM2 inhibition has differential effects on CD8^+^ T-cell subset viability

Because p53 has been shown to exert context-dependent (35) effects on apoptosis and cell-cycle arrest, we next examined whether the expansion of the central memory (Tcm) subset relative to the effector memory (Tem) subset in activated CD8^+^ T cells was associated with differences in viability. This will also help us determine if Tcm expansion was not an artifact of Tem reduction by treatment with an MDM2 inhibitor. At concentrations of 1μM and lower, Navtemadlin maintained similar numbers of live cells and culture viability while increasing the number of central memory T cells and modestly reducing the number of effector memory compared to control cultures (**Figures 3A, B**). Further analysis with propidium iodide and annexin V revealed Navtemadlin treatment increased the proportion of viable, non-apoptotic cells (annexin V- and PI -) within the Tcm compartment while modestly reducing the viable, non-apoptotic cell population in Tem at 1μM concentration (**Figure 3C**). At concentration of 2μM, however, both the numbers of Tcm and Tem (**Figure 3B**) along with their proportions of viable, non-apoptotic cells (**Supplementary Figures S2A, B**) sharply decreased, suggesting drug toxicity towards both T-cell subsets rather than just Tem when used at concentrations higher than 1μM.

**Figure 3:**
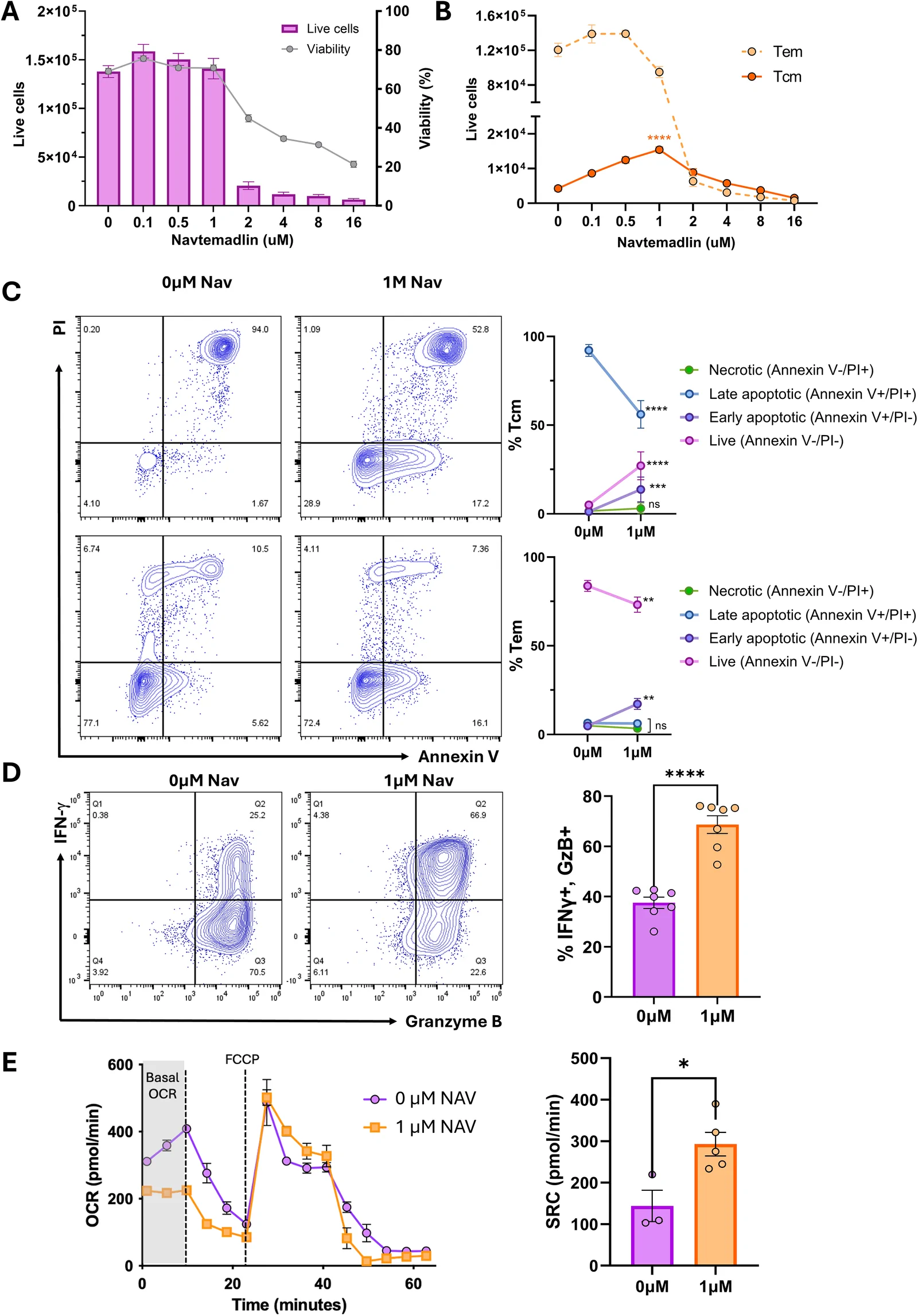
MDM2 mitigates apoptosis in central memory T cells and enhances metabolic fitness and polyfunctionality. Naïve human CD8+ T cells were activated with CD3/CD28/IL-2 for 5 days at different concentration of Navtemadlin as indicated. **(A)** Number of total live cells and viability and **(B)** number of live central and effector memory (right) were shown (n ≥ 3 biological replicates). **(C)** Representative Annexin V and PI flow plots for central (top) and effector (bottom) memory at 0μM and 1μM of Navtemadlin on day 5. Frequency of live (Annexin V- PI-), early apoptotic (Annexin V+ PI-), late apoptotic (Annexin V+ PI+), and necrotic (Annexin V- PI+) were plotted as seen in the bar plots (n = 3 biological replicates). **(D)** Representative flow plots of granzyme B (GzB) and interferon-gamma (IFN-γ) at 0μM and 1μM of Navtemadlin after 5-day culture (left). Frequency of polyfunctional CD8+ T cells were quantified (right) (n ≥ 3 biological replicates). **(E)** Oxygen consumption rate (OCR) and spare respiratory capacity (SRC) at 0μM and 1 μM of Navtemadlin (n ≥ 3 biological replicates). Oligomycin was injected after the 3rd measurement (approximately at minute 12), FCCP after the 6th measurement (approximately at minute 25), and rotenone and antimycin A after the 10th measurement (approximately at minute 40). Asterisks indicate statistical significance by one-way ANOVA followed by Dunnett’s multiple comparisons test: *p < 0.05, **p < 0.01, ***p < 0.001, ****p < 0.0001. Bars represent mean and error bars indicate SEM. Colored asterisk indicate significant difference compared to 0 μM NAV control.

Additionally, Navtemadlin treatment at 1μM reduced the number of terminally differentiated effector memory T cells (TEMRA) compared to cultured controls (**Supplementary Figure S2C, D**). Given these findings, p53 activation can be optimized to selectively promote the survival and expansion of memory-like CD8+ T cells while constraining the persistence of more differentiated effector memory populations.

### p53 activation enhances metabolic fitness and polyfunctionality

Having observed increased expression of effector-associated genes, including interferon-γ (IFNG) and granzyme B (GZMB), in the transcriptional analysis (**Figure 2B**), we next evaluated whether Navtemadlin treatment impacted CD8^+^ T-cell functional responses. Functional analysis revealed that Navtemadlin-treated CD8^+^ T cells displayed increased frequencies of polyfunctional cells producing both interferon-γ and granzyme B compared with untreated controls (**Figure 3C**). Because mitochondrial metabolism plays a critical role in sustaining T-cell function (36–38), we next assessed the metabolic fitness of treated cells. Metabolic profiling demonstrated that Navtemadlin-treated CD8^+^ T cells exhibited significantly increased mitochondrial spare respiratory capacity (SRC) with reduced basal respirator demand (**Figure 3D**; **Supplementary Figure S3**), a hallmark feature associated with long-lived memory T-cell responses and enhanced effector potential. Increased SRC reflects an improved ability of mitochondria to generate additional energy in response to cellular stress or activation, a property that supports sustained cytotoxic function in memory CD8^+^ T cells (39). Collectively, these findings indicate that pharmacologic activation of the p53 pathway enhances both the functional capacity and metabolic fitness of CD8^+^ T cells.

### p53 activation improves antitumor activity of engineered T cells

To evaluate the translational relevance of p53 activation in CD8+ T cells, we generated tumor-specific TCR-engineered CD8+ T cells in the presence or absence of the MDM2 inhibitor Navtemadlin. Transient pharmacologic activation of p53 concurrently with T-cell activation, followed by TCR transduction, produced engineered T cells with enhanced functional capacity. Prior to tumor co-culture, Navtemadlin-treated TCR-engineered CD8+ T cells showed increased frequencies of polyfunctional cells co-expressing interferon-γ and granzyme B compared with untreated controls, as expected (**Figure 4A**).

**Figure 4:**
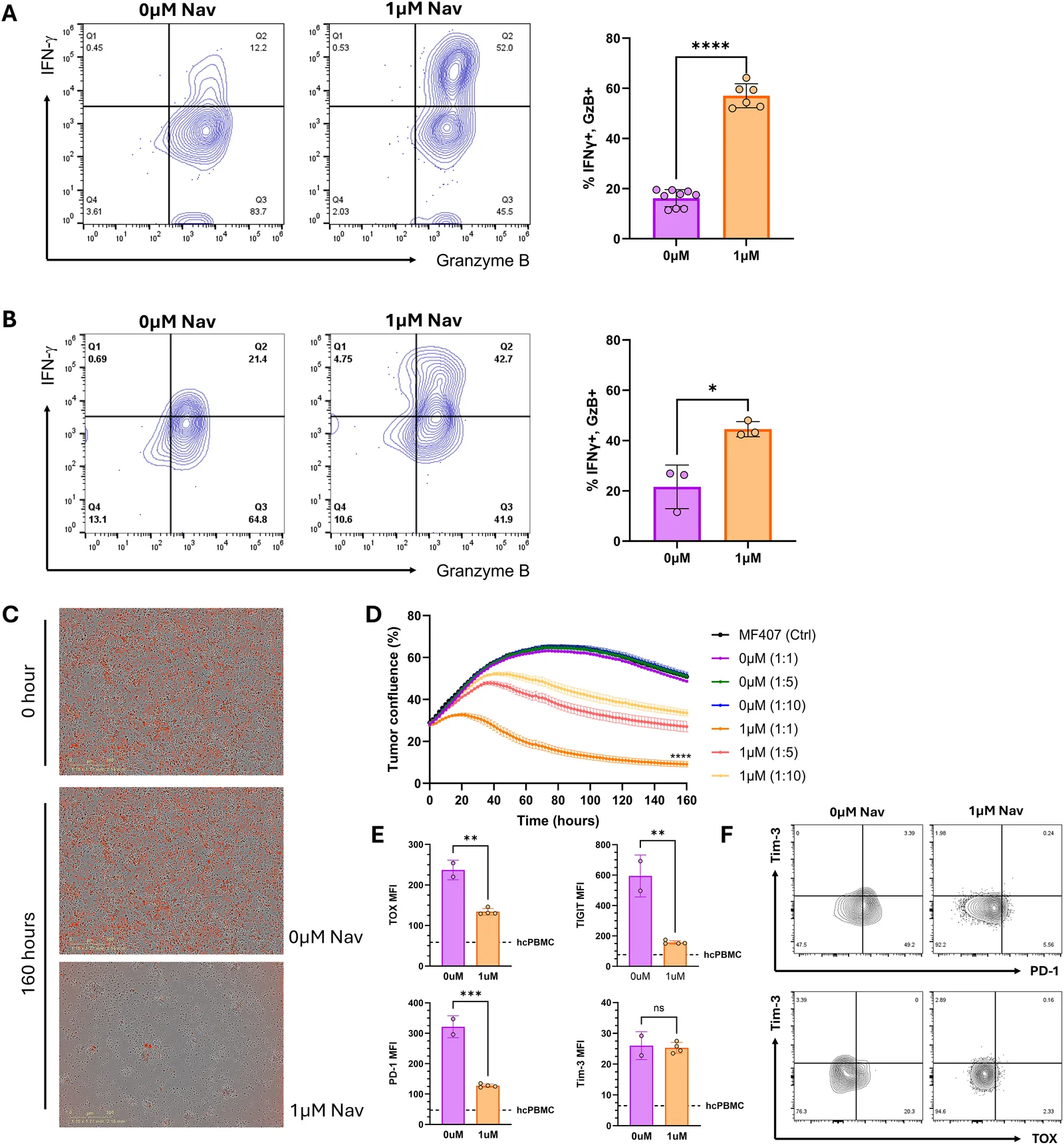
MDM2 inhibition constrains functional exhaustion and improves anti-tumor immunity of MAGE-A4 directed CD8 T cells. Isolated human naïve CD8+ T cells from healthy donors were activated with CD3/CD28/IL-2 for 5 days with or without 1µM of Navtemadlin. Tumor-specific MAGE-A4 TCR was transduced to CD8+ T cells on day 2. On day 6, TCR-engineered T cells were co-cultured with MAGE-A4 positive melanoma cells (M407) without additional use of Navtemadlin. Different ratios of effectors (MAGE-A4 TCR+ CD8+ T cells)-to-target (M407 melanoma) were tested, including 1:1, 1:5, and 1:10. Cytotoxic functions of CD8+ T cells were measured and quantified before **(A)** and after **(B)** co-culture with M407 melanoma (n ≥ 2 biological replicates). **(C)** Representative images of the co-culture before and after the tumor-killing assay show increased clearing of tumor cells (red dots) by TCR-engineered T cells that were previously treated with 1 µM of Navtemadlin. **(D)** Previously NAV-treated CD8+ T cells showed increased tumor clearing across different effector-to-target (E:T) ratios (n = 3 biological replicates). **(E)** TOX, TIGIT, PD-1 and Tim-3 MFI values were measured after the co-culture when gating on CD8+ and MAGE-A4 TCR+ cells at 1:1 E:T ratio. **(F)** Representative flow plots showing PD-1, TIM-3, and TOX expression gated on live CD8+ TCR+ cells. Unpaired T-test was performed, and statistical significance is indicated as follows: *p < 0.05, **p < 0.01, ***p < 0.001, ****p < 0.0001. Bars represent mean and error bars indicate SEM.

We then next assessed antitumor activity using antigen-specific tumor-killing assays. MAGE-A4–directed TCR-engineered CD8+ T cells were co-cultured against MAGE-4 expressing melanoma cells in the absence of IL-2, since translational efforts increasingly aim to improve T-cell persistence *in vivo* without relying on IL-2 post adoptive cell transfer as IL-2 can drive terminal differentiation and systemic toxicity (40). Consistent with improved functional durability, Navtemadlin-conditioned TCR-engineered CD8+ T cells maintained higher frequencies of polyfunctional interferon-γ and granzyme B producing cells after 5 days of co-culture (**Figure 4B**). Longitudinal live-cell imaging revealed accelerated tumor clearance beginning at 40 hours over a 160-hour time course, relative to control T cells and across multiple effector-to-target ratios (**Figures 4C, D**). As expected, control cells cleared tumor cells poorly under these IL-2 free conditions. Navtemadlin-treated cells also showed reduced expression (by MFI) of the exhaustion-associated markers TOX, TIGIT, and PD-1 (**Figure 4E**), while higher percentages of PD-1+, TIM-3+, and TOX+ cells were observed in the untreated group (**Figure 4F**).

Consistent with these findings, Navtemadlin-treated TCR-engineered CD8+ T cells restimulated with CD3/CD28 in the absence of IL-2 showed reduced expression of additional exhaustion-associated markers, including the ectonucleotidase CD39 and the co-inhibitory receptors LAG-3 and CTLA-4 (**Supplementary Figures S4A, B**). Navtemadlin-treated cells also displayed higher proliferative capacity than untreated controls (**Supplementary Figure S4C**). Together, these findings suggest that transient pharmacologic activation of the p53 pathway during T-cell manufacturing may enhance the durability and therapeutic potency of adoptive T-cell therapies, even in the absence of IL-2 support.

## Discussion

This study identifies transient pharmacologic activation of the p53 pathway as a potential strategy to enhance the functional quality of human CD8+ T cells during ex vivo manufacturing. Although p53 has traditionally been viewed primarily as a tumor suppressor that regulates cell-cycle arrest and apoptosis, our findings support an additional role for p53 in shaping T-cell differentiation, survival, and metabolic fitness. Pharmacologic activation of p53 through MDM2 inhibition promoted the formation of less differentiated memory T-cell subsets while limiting terminal effector differentiation, suggesting that transient p53 activation may help preserve T-cell states associated with improved therapeutic potential.

At the optimal concentration tested, 1μM of Navtemadlin significantly increased the proportion of central memory T cells while modestly reducing the effector memory population and limiting the generation of terminally differentiated effector memory cells. The enrichment of central memory or stem central memory T cells is particularly relevant for adoptive cell therapy because these cells exhibit greater proliferative capacity upon antigen re-encounter and have been associated with enhanced persistence antitumor activity (2–4). In our experiments with TCR-engineered CD8+ T cells, we found Navtemadlin-treated cells exhibited a higher proliferative capacity compared to untreated cells when restimulated with CD3/CD28 in the absence of exogenous IL-2. Moreover, transient exposure to Navtemadlin during early T-cell activation did not compromise overall cellular expansion, suggesting that pharmacologic p53 modulation could potentially be incorporated into existing ex vivo manufacturing workflows to improve the quality of T-cell products without substantially impairing the generation of sufficient cell numbers.

The preservation of a less differentiated phenotype was accompanied by reduced expression of exhaustion-associated genes following T-cell activation, further supporting the potential application of Navtemadlin during the manufacture of engineered T-cell products. Conventional CAR-T cell manufacturing frequently relies on IL-2 to support T-cell survival and proliferation (41); however, prolonged stimulation and sustained exposure to high concentrations of IL-2 can promote terminal effector differentiation and may compromise the long-term functional properties of the resulting cell product (42). Consistent with this concept, we observed substantial loss of central memory T cells in control cultures, whereas Navtemadlin treatment increased the number of viable central memory T cells and importantly preserved a larger population of healthy, non-apoptotic cells. Alternative manufacturing strategies have therefore incorporated cytokines such as IL-7, IL-15, and IL-21 to promote the generation or maintenance of memory-associated T-cell states (41, 43). Our findings raise the possibility that transient p53 activation may provide an additional, mechanistically distinct approach to preserving desirable T-cell phenotypes during manufacturing.

Metabolic fitness represents another important determinant of engineered T-cell function (39). In particular, increased mitochondrial mass, spare respiratory capacity (SRC), and metabolic flexibility between glycolysis and oxidative phosphorylation have been associated with improved T-cell persistence and function (44). PGC-1a-dependent regulation of mitochondrial biogenesis and oxidative metabolism has been extensively studied in this context (45). Our findings suggest that p53 activation may represent another pathway capable of influencing mitochondrial fitness in human CD8+ T cells. Mechanistically, p53 has been reported to promote mitochondrial oxidative phosphorylation through regulation of TP53-induced glycolysis regulator (TIGAR) and synthesis of cytochrome c oxidase (SCO2) while also restricting glycolytic metabolism through regulation of glucose transporters such as GLUT1 and GLUT4 (46). These effects may provide a potential mechanistic framework for further studies of the improved spare respiratory capacity observed in CD8+ T cells following Navtemadlin treatment.

Importantly, p53 activity must be appropriately controlled because sustained or excessive p53 activation can have opposing effects on cell survival. Strong or prolonged p53 signaling can induce pro-apoptotic mediators including PUMA, NOXA, and BAX, leading to permeabilization of mitochondrial outer membrane and apoptosis (47). Thus, the beneficial effects of p53 activation may depend on the magnitude and duration of pathway activation. Navtemadlin has a reported plasma half-life of approximately 18 hours in human (48), and its transient incorporation at the initiation of T-cell culture may provide an initial period of p53 activation followed by declining pharmacologic exposure. Whether this temporal pattern is responsible for separating the beneficial metabolic and differentiation effects of p53 activation from its pro-apoptotic effects remains to be determined. Nevertheless, these findings support the concept that carefully controlled, transient p53 modulation may improve T-cell fitness while avoiding excessive activation of the apoptotic program.

Our results also demonstrate clear translational potential. TCR-engineered CD8+ T cells generated in the presence of Navtemadlin displayed enhanced tumor-killing capacity compared with conventionally manufactured cells. Because MDM2 inhibitors are already being developed clinically, transient pharmacologic modulation of the p53 pathway represents a feasible approach to improve engineered T-cell products without requiring additional genetic modification.

The effects of p53 modulation on CD4+ T cells also warrant investigation. Although the present study focused primarily on CD8+ T cells, CD4+ and CD8+ T cells share many fundamental signaling pathways involved in T-cell activation and proliferation, including TCR/CD3 signaling, CD28-mediated co-stimulation, and IL-2-STAT5 signaling (49). It is therefore plausible that p53 modulation could influence CD4+ T-cell differentiation following initial activation; however, CD4+ T-cell effector functions will differ by phenotype, and additional studies will be required. Determining whether Navtemadlin similarly preserves memory-associated CD4+ T-cell populations could be relevant for engineered T-cell products in which both CD4+ and CD8+ T cells contribute to antitumor activity.

Several limitations should be acknowledged. First, the precise molecular mechanisms by which p53 influences T-cell fate remain incompletely understood, and future studies will be required to define the downstream pathways responsible for these effects. Second, although Navtemadlin-treated TCR-engineered CD8+ T cells demonstrated enhanced tumor-killing capacity, control cells exhibited relatively limited tumor killing despite the presence of exhaustion-associate phenotypes. Another possible explanation is that Navtemadlin-treated cells may have reduced dependence on exogenous IL-2 or retained greater functional fitness under conditions of limited cytokine availability. This possibility is supported by the observation that treated cells proliferated more than untreated cells during tumor co-culture or restimulation in the absence of exogenous IL-2. However, this mechanism remains hypothetical and will require direct assessment of cytokine dependence. Finally, *in vivo* studies will be necessary to determine whether the enhanced functional properties observed *in vitro* translate to improved persistence and therapeutic efficacy after adoptive transfer.

## Conclusion

Overall, our findings identify transient pharmacologic activation of the p53 pathway as a potential strategy to improve the functional properties of engineered CD8+ T cells. MDM2 inhibition promoted a less differentiated, memory-like state accompanied by reduced exhaustion-associated features, improved metabolic fitness and polyfunctionality, and enhanced antitumor activity. These findings suggest controlled modulation of the MDM2-p53 axis could be incorporated into engineered T-cell manufacturing to generate products with greater persistence and therapeutic efficacy. Further mechanistic and *in vivo* studies are needed to determine the optimal dose and duration of p53 activation required to maximize T-cell fitness while avoiding p53-dependent apoptosis.

## Data Availability

The data presented in the study are deposited in the NCBI Gene Expression Omnibus (GEO) repository, accession number GSE350216.

## Supplementary material

Supplementary Figure 1 Isolated naïve human CD8+ T cells were activated with CD3 and CD28 antibodies in the presence of IL-2 for 5 days. **(A)** Expression of p53+ when gated on each live subsets of CD3+ CD8+ T cells, including naïve, stem central memory (Tscm), central memory (Tcm), effector memory (Tem), effector (Teff), and terminally differentiated effector memory (TEMRA) T cells (n = 3 biological replicates). **(B)** Quantification of p53 expression and MFI based on each subset (n = 3 biological replicates). **(C)** Normalized p53 MFI of CD3+ CD8+ T cells in culture overtime at indicated concentrations of Navtemadlin in the presence of IL-2 with and without TCR stimulation using CD3 and CD28 antibodies (n = 3 biological replicates).

Supplementary Figure 2 Isolated naïve human CD8+ T cells were activated with CD3 and CD28 antibodies in the presence of IL-2 for 5 days. **(A)** Annexin V and PI analysis of CD3+ CD8+ T cells when gated on central memory (Tcm) and effector memory (Tem) (n = 3 biological replicates). Frequency of live (Annexin V- PI-), early apoptotic (Annexin V+ PI-), late apoptotic (Annexin V+ PI+), and necrotic (Annexin V- PI+) were plotted as shown in the representative flow plots **(B)**. **(C)** Quantification of CD8+ T-cell subsets in culture when gated on live CD3+ CD8+ T cells (n = 3 biological replicates) along with the representative flow plots (D). Top row is gated on live CD3+ CD8+ while bottom row is further gated on CCR7- and CD45RO-.

Supplementary Figure 3 Basal, ATP-linked, and maximal oxygen consumption rate (OCR) in addition to the spare reserve capacity at 0μM and 1μM of Navtemadlin (n ≥ 3 biological replicates).

Supplementary Figure 4 Isolated naïve human CD8+ T cells were activated with CD3 and CD28 dynabeads in the presence of IL-2 for 5 days with or without 1μM of Navtemadlin. Fresh media was added on day 2 to mimic TCR transduction step. On day 6, cells from each condition were then transferred into fresh media containing new CD3/CD28 dynabeads but no added IL-2 and cultured for an additional 5 days. All steps and cell numbers used for the manufacturing of TCR-engineered T cells and the tumor co-culture were kept identical. **(A)** MFI values of ehaustion-associated markers were quantified on day 6 before T-cell restimulation and on day 10. **(B)** Representative flow plots showed T-cell expression of different exhaustion markers on day 10 when gated on live CD3+ and CD8 +. **(C)** Number of live cells between Navtemadlin-treated and untreated conditions when seeded at the same number of cells on day 6 (n = 4 technical replicates).

## References

[1] Srour SA Akin S . Chimeric antigen receptor T-cell therapy for solid tumors: The past and the future. J Immunother Precis Oncol. (2023) 6:19–30. doi: 10.36401/JIPO-22-7 PMC9888521 36751657

[2] Klebanoff CA Scott CD Leonardi AJ Yamamoto TN Cruz AC Ouyang C . Memory T cell–driven differentiation of naive cells impairs adoptive immunotherapy. J Clin Invest. (2016) 126:318–34. doi: 10.1172/JCI81217 PMC4701537 26657860

[3] Busch DH Fräßle SP Sommermeyer D Buchholz VR Riddell SR . Role of memory T cell subsets for adoptive immunotherapy. Semin Immunol. (2016) 28:28–34. doi: 10.1016/j.smim.2016.02.001 26976826 PMC5027130

[4] McLellan AD Ali Hosseini Rad SM . Chimeric antigen receptor T cell persistence and memory cell formation. Immunol Cell Biol. (2019) 97:664–74. doi: 10.1111/imcb.12254 31009109

[5] Klebanoff CA Crompton JG Leonardi AJ Yamamoto TN Chandran SS Eil RL . Inhibition of AKT signaling uncouples T cell differentiation from expansion for receptor-engineered adoptive immunotherapy (2017). Available online at: https://insight.jci.org/articles/view/95103 (Accessed August 30, 2026). 10.1172/jci.insight.95103 PMC5752304 29212954

[6] Crompton JG Sukumar M Restifo NP . Uncoupling T-cell expansion from effector differentiation in cell-based immunotherapy. Immunol Rev. (2014) 257:264–76. doi: 10.1111/imr.12135 PMC3915736 24329803

[7] Crompton JG Sukumar M Roychoudhuri R Clever D Gros A Eil RL . Akt inhibition enhances expansion of potent tumor-specific lymphocytes with memory cell characteristics. Cancer Res. (2015) 75:296–305. doi: 10.1158/0008-5472.can-14-2277 25432172 PMC4384335

[8] Kaiser AM Attardi LD . Deconstructing networks of p53-mediated tumor suppression *in vivo*. Cell Death Differ. (2018) 25:93–103. doi: 10.1038/cdd.2017.171 PMC5729531 29099489

[9] Vousden KH Lane DP . p53 in health and disease. Nat Rev Mol Cell Biol. (2007) 8:275–83. doi: 10.1038/nrm2147 17380161

[10] Wu X Bayle JH Olson D Levine AJ . The p53-mdm-2 autoregulatory feedback loop. Genes Dev. (1993) 7:1126–32. doi: 10.1101/gad.7.7a.1126 8319905

[11] Shangary S Wang S . Targeting the MDM2-p53 interaction for cancer therapy. Clin Cancer Res Off J Am Assoc Cancer Res. (2008) 14:5318–24. doi: 10.1158/1078-0432.ccr-07-5136 18765522 PMC2676446

[12] Koo N Sharma AK Narayan S . Therapeutics targeting p53-MDM2 interaction to induce cancer cell death. Int J Mol Sci. (2022) 23:5005. doi: 10.3390/ijms23095005 35563397 PMC9103871

[13] Zhu H Gao H Ji Y Zhou Q Du Z Tian L . Targeting p53–MDM2 interaction by small-molecule inhibitors: Learning from MDM2 inhibitors in clinical trials. J Hematol Oncol. (2022) 15:91. doi: 10.1186/s13045-022-01314-3 35831864 PMC9277894

[14] Lerma Clavero A Boqvist PL Ingelshed K Bosdotter C Sedimbi S Jiang L . MDM2 inhibitors, nutlin-3a and navtemadelin, retain efficacy in human and mouse cancer cells cultured in hypoxia. Sci Rep. (2023) 13:4583. doi: 10.1038/s41598-023-31484-0 36941277 PMC10027891

[15] Tolcher AW Reeves JA McKean M Chmielowski B Beck JT Shaheen MF . Preliminary results of a phase II study of alrizomadlin (APG-115), a novel, small-molecule MDM2 inhibitor, in combination with pembrolizumab in patients (pts) with unresectable or metastatic melanoma or advanced solid tumors that have failed immuno-oncologic (I-O) drugs. J Clin Oncol. (2021) 39:2506. doi: 10.1200/jco.2021.39.15_suppl.250642697810

[16] Zhang X Wen X Peng R Pan Q Weng D Ma Y . A first-in-human phase I study of a novel MDM2/p53 inhibitor alrizomadlin in advanced solid tumors (2024). Available online at: https://www.esmoopen.com/article/S2059-7029(24)01405-4/fulltext (Accessed May 26, 2026). 10.1016/j.esmoop.2024.103636 PMC11452328 39002360

[17] Fang DD Tang Q Kong Y Rong T Wang Q Li N . MDM2 inhibitor APG-115 exerts potent antitumor activity and synergizes with standard-of-care agents in preclinical acute myeloid leukemia models. Cell Death Discov. (2021) 7:90. doi: 10.1038/s41420-021-00465-5 33941774 PMC8093284

[18] Rendo V Lee EQ Bossi C Khuu N Rudek MA Pal S . Surgical window of opportunity trial reveals mechanisms of response and resistance to navtemadlin (KRT-232) in patients with recurrent glioblastoma (2024) (Accessed May 26, 2026). preprint. 10.1126/scitranslmed.adn6274 PMC11994224 39970230

[19] Dudley ME Wunderlich JR Shelton TE Even J Rosenberg SA . Generation of tumor-infiltrating lymphocyte cultures for use in adoptive transfer therapy for melanoma patients. J Immunother. (2003) 26:332–42. doi: 10.1097/00002371-200307000-00005 12843795 PMC2305721

[20] Divakaruni AS Rogers GW Murphy AN . Measuring mitochondrial function in permeabilized cells using the Seahorse XF Analyzer or a Clark-type oxygen electrode. Curr Protoc Toxicol. (2014) 60:25.2.1–16. doi: 10.1002/0471140856.tx2502s60 24865646

[21] Desousa BR Kim KK Jones AE Ball AB Hsieh WY Swain P . Calculation of ATP production rates using the Seahorse XF Analyzer. EMBO Rep. (2023) 24:e56380. doi: 10.15252/embr.202256380 37548091 PMC10561364

[22] Chang C-H Pearce EL . Emerging concepts of T cell metabolism as a target of immunotherapy. Nat Immunol. (2016) 17:364–8. doi: 10.1038/ni.3415 27002844 PMC4990080

[23] Chapman NM Boothby MR Chi H . Metabolic coordination of T cell quiescence and activation. Nat Rev Immunol. (2020) 20:55–70. doi: 10.1038/s41577-019-0203-y 31406325

[24] Liu Y Elf SE Miyata Y Sashida G Liu Y Huang G . p53 regulates hematopoietic stem cell quiescence. Cell Stem Cell. (2009) 4:37–48. doi: 10.1016/j.stem.2008.11.006 19128791 PMC2839936

[25] Watanabe M Moon KD Vacchio MS Hathcock KS Hodes RJ . Downmodulation of tumor suppressor p53 by T cell receptor signaling is critical for antigen-specific CD4+ T cell responses. Immunity. (2014) 40:681–91. doi: 10.1016/j.immuni.2014.04.006 24792911 PMC4073799

[26] Bourdon J . p53 isoforms change p53 paradigm. Mol Cell Oncol. (2014) 1:e969136. doi: 10.4161/23723548.2014.969136 27308370 PMC4905211

[27] Marcel V Perrier S Aoubala M Ageorges S Groves MJ Diot A . Δ160p53 is a novel N-terminal p53 isoform encoded by Δ133p53 transcript. FEBS Lett. (2010) 584:4463–8. doi: 10.1016/j.febslet.2010.10.005 20937277

[28] Flaman JM Waridel F Estreicher A Vannier A Limacher JM Gilbert D . The human tumour suppressor gene p53 is alternatively spliced in normal cells. Oncogene. (1996) 12:813–8. 8632903

[29] Anbarasan T Bourdon J-C . The emerging landscape of p53 isoforms in physiology, cancer and degenerative diseases. Int J Mol Sci. (2019) 20:6257. doi: 10.3390/ijms20246257 31835844 PMC6941119

[30] Joruiz SM Bourdon J-C . p53 isoforms: Key regulators of the cell fate decision. Cold Spring Harb Perspect Med. (2016) 6:a026039. doi: 10.1101/cshperspect.a026039 26801896 PMC4968168

[31] Khoury MP Bourdon J-C . p53 isoforms. Genes Cancer. (2011) 2:453–65. doi: 10.1177/1947601911408893 PMC3135639 21779513

[32] Steffens Reinhardt L Zhang X Groen K Morten BC De Iuliis GN Braithwaite AW . Alterations in the p53 isoform ratio govern breast cancer cell fate in response to DNA damage. Cell Death Dis. (2022) 13:907. doi: 10.1038/s41419-022-05349-9 36307393 PMC9616954

[33] Neitzke-Montinelli V Calôba C Melo G Frade BB Caramez E Mazzoccoli L . Differentiation of memory CD8 T cells unravel gene expression pattern common to effector and memory precursors. Front Immunol. (2022) 13:840203. doi: 10.3389/fimmu.2022.840203 35677061 PMC9168330

[34] Chu T Wu M Hoellbacher B de Almeida GP Wurmser C Berner J . Precursors of exhausted T cells are pre-emptively formed in acute infection. Nature. (2025) 640:782–92. doi: 10.1038/s41586-024-08451-4 39778709 PMC12003159

[35] Targeting Cellular Senescence in Cancer and Aging: Roles of P53 and its Isoforms | Carcinogenesis | Oxford Academic. Available online at: https://academic.oup.com/carcin/article/41/8/1017/5867012?login=true (Accessed May 26, 2026). 10.1093/carcin/bgaa071 PMC7422622 32619002

[36] van der Windt GJW O’Sullivan D Everts B Huang S-C Buck MD Curtis JD . CD8 memory T cells have a bioenergetic advantage that underlies their rapid recall ability. Proc Natl Acad Sci. (2013) 110:14336–41. doi: 10.1073/pnas.1221740110 23940348 PMC3761631

[37] Sukumar M Liu J Ji Y Subramanian M Crompton JG Yu Z . Inhibiting glycolytic metabolism enhances CD8+ T cell memory and antitumor function. J Clin Invest. (2013) 123:4479–88. doi: 10.1172/jci69589 24091329 PMC3784544

[38] Sukumar M Liu J Mehta GU Patel SJ Roychoudhuri R Crompton JG . Mitochondrial membrane potential identifies cells with enhanced stemness for cellular therapy. Cell Metab. (2016) 23:63–76. doi: 10.1016/j.cmet.2015.11.002 26674251 PMC4747432

[39] Rad SMA Halpin JC Mollaei M Smith Bell SWJ Hirankarn N McLellan AD . Metabolic and mitochondrial functioning in chimeric antigen receptor (CAR)—T cells. Cancers. (2021) 13:1229. doi: 10.3390/cancers13061229 PMC8002030 33799768

[40] Guo J Wang C Luo N Wu Y Huang W Zhu J . IL-2-free tumor-infiltrating lymphocyte therapy with PD-1 blockade demonstrates potent efficacy in advanced gynecologic cancer. BMC Med. (2024) 22:207. doi: 10.1186/s12916-024-03420-0 38769543 PMC11106999

[41] Guaza-Lasheras M Nimmerfroh J Schwarz D de Gruijl TD Hartmann J Klein C . The evolving role of cytokines for CAR-T cell manufacturing and beyond. Nat BioMed Eng. (2026) 10(9):1811–27. doi: 10.1038/s41551-026-01703-w 42373802 PMC7619229

[42] Zhu X Li Q Zhu X . Mechanisms of CAR T cell exhaustion and current counteraction strategies (2022). Frontiers. Available online at: https://www.frontiersin.org/journals/cell-and-developmental-biology/articles/10.3389/fcell.2022.1034257/full (Accessed August 28, 2026). 10.3389/fcell.2022.1034257 PMC9773844 36568989

[43] Tan JT Ernst B Kieper WC LeRoy E Sprent J Surh CD . Interleukin (IL)-15 and IL-7 jointly regulate homeostatic proliferation of memory phenotype CD8+ cells but are not required for memory phenotype CD4+ cells. J Exp Med. (2002) 195:1523–32. doi: 10.1084/jem.20020066 12070280 PMC2193564

[44] DePeaux K Delgoffe GM . Metabolic barriers to cancer immunotherapy. Nat Rev Immunol. (2021) 21:785–97. doi: 10.1038/s41577-021-00541-y 33927375 PMC8553800

[45] Van der Vreken A Vanderkerken K De Bruyne E De Veirman K Breckpot K Menu E . Fueling CARs: Metabolic strategies to enhance CAR T-cell therapy. Exp Hematol Oncol. (2024) 13:66. doi: 10.1186/s40164-024-00535-1 38987856 PMC11238373

[46] Zhang X Qin Z Wang J . The role of p53 in cell metabolism. Acta Pharmacol Sin. (2010) 31:1208–12. doi: 10.1038/aps.2010.151 20729871 PMC4002322

[47] Flöter J Kaymak I Schulze A . Regulation of metabolic activity by p53. Metabolites. (2017) 7:21. doi: 10.3390/metabo7020021 PMC5487992 28531108

[48] Pharmacokinetics and Macrophage Inhibitory Cytokine‐1 Pharmacodynamics of the Murine Double Minute 2 Inhibitor, Navtemadlin (KRT‐232) in Fed and Fasted Healthy Subjects - Wong - 2022 - Clinical Pharmacology in Drug Development - Wiley Online Library (Accessed August 28, 2026). 10.1002/cpdd.1070 PMC9306949 35172043

[49] Seder RA Ahmed R . Similarities and differences in CD4+ and CD8+ effector and memory T cell generation. Nat Immunol. (2003) 4:835–42. doi: 10.1038/ni969 12942084

