# Supplementary material for: p53 activation via pharmacologic MDM2 inhibition constrains exhaustion and enhances antitumor function of human CD8+ T cells
Source: Front Immunol. 2026 Oct 16;17:1910235. doi: 10.3389/fimmu.2026.1910235 (PMC13719607; doi:10.3389/fimmu.2026.1910235)
Supplement: Supplementary file 1 [file DataSheet1.pdf]

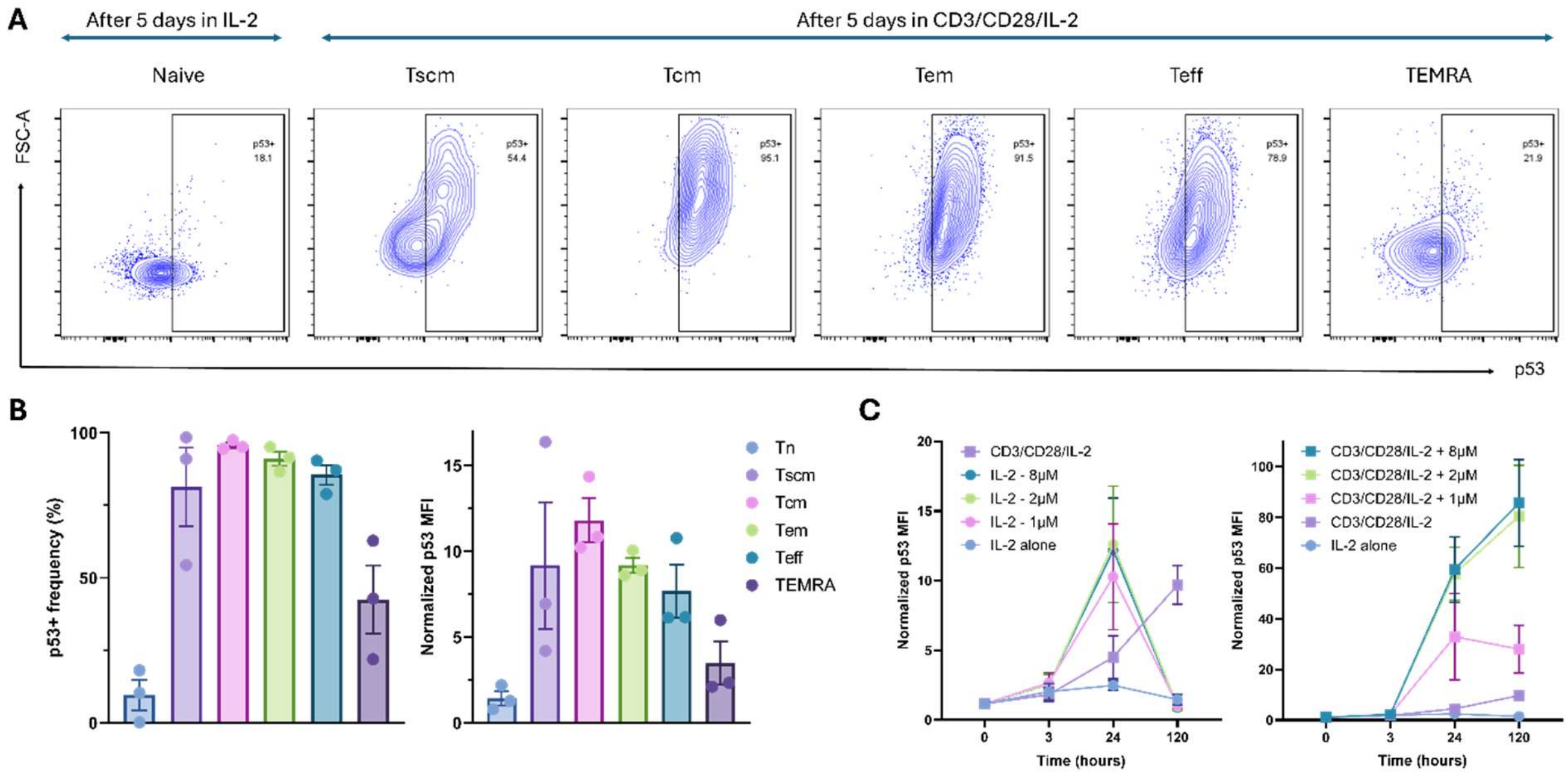

Supplementary Figure S1: Isolated naïve human CD8+ T cells were activated with CD3 and CD28 antibodies in the presence of IL-2 for 5 days. (A) Expression of p53+ when gated on each live subsets of CD3+ CD8+ T cells, including naïve, stem central memory (Tscm), central memory (Tcm), effector memory (Tem), effector (Teff), and terminally differentiated effector memory (TEMRA) T cells (n = 3 biological replicates). (B) Quantification of p53 expression and MFI based on each subset (n = 3 biological replicates). (C) Normalized p53 MFI of CD3+ CD8+ T cells in culture overtime at indicated concentrations of Navtemadlin in the presence of IL-2 with and without TCR stimulation using CD3 and CD28 antibodies (n = 3 biological replicates).

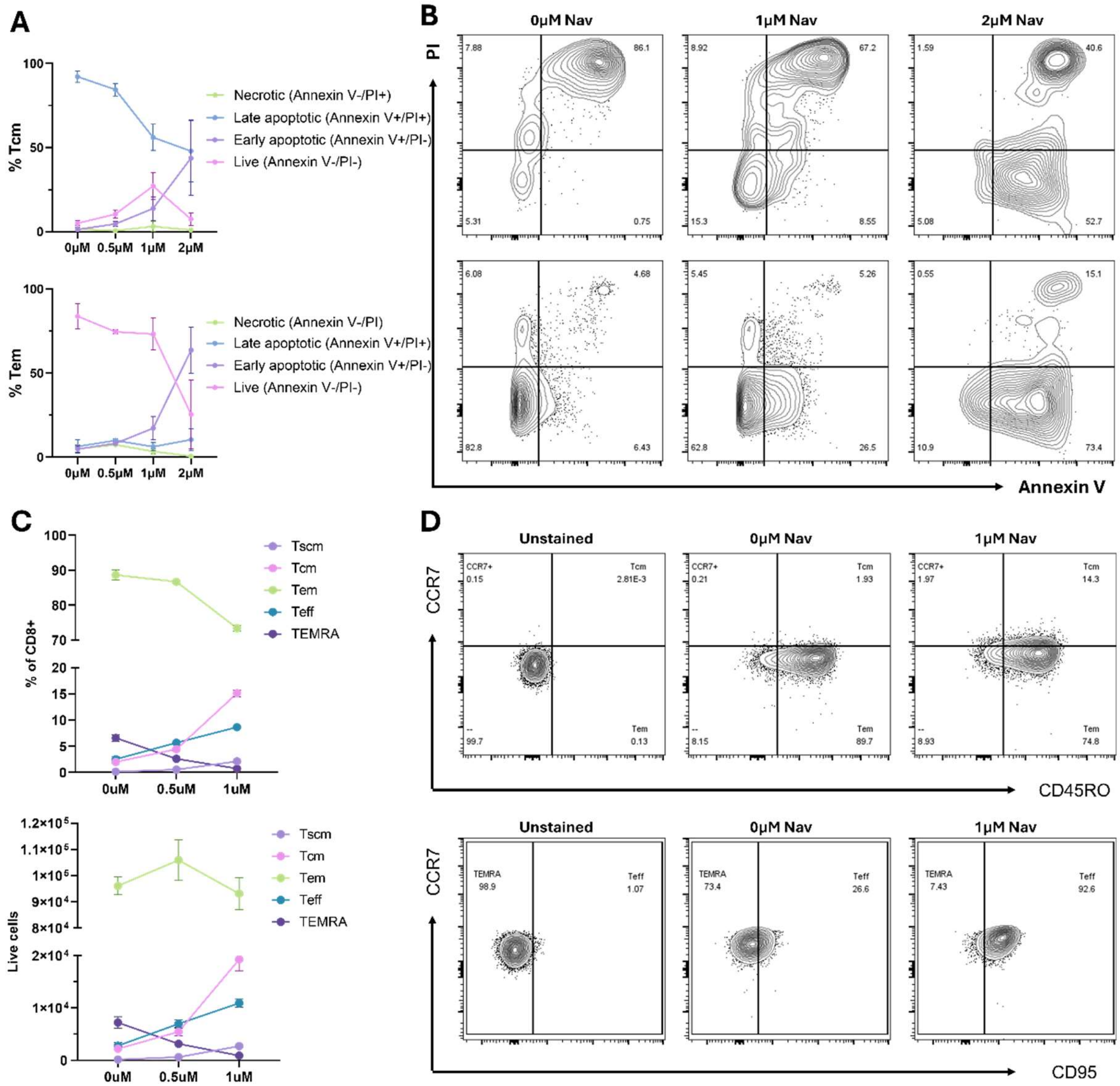

Supplementary Figure S2: Isolated naïve human CD8+ T cells were activated with CD3 and CD28 antibodies in the presence of IL-2 for 5 days. (A) Annexin V and PI analysis of CD3+ CD8+ T cells when gated on central memory (Tcm) and effector memory (Tem) (n = 3 biological replicates). Frequency of live (Annexin V- PI-), early apoptotic (Annexin V+ PI-), late apoptotic (Annexin V+ PI+), and necrotic (Annexin V- PI+) were plotted as shown in the representative flow plots (B). (C) Quantification of CD8+ T-cell subsets in culture when gated on live CD3+ CD8+ T cells (n = 3 biological replicates) along with the representative flow plots (D). Top row is gated on live CD3+ CD8+ while bottom row is further gated on CCR7- and CD45RO-.

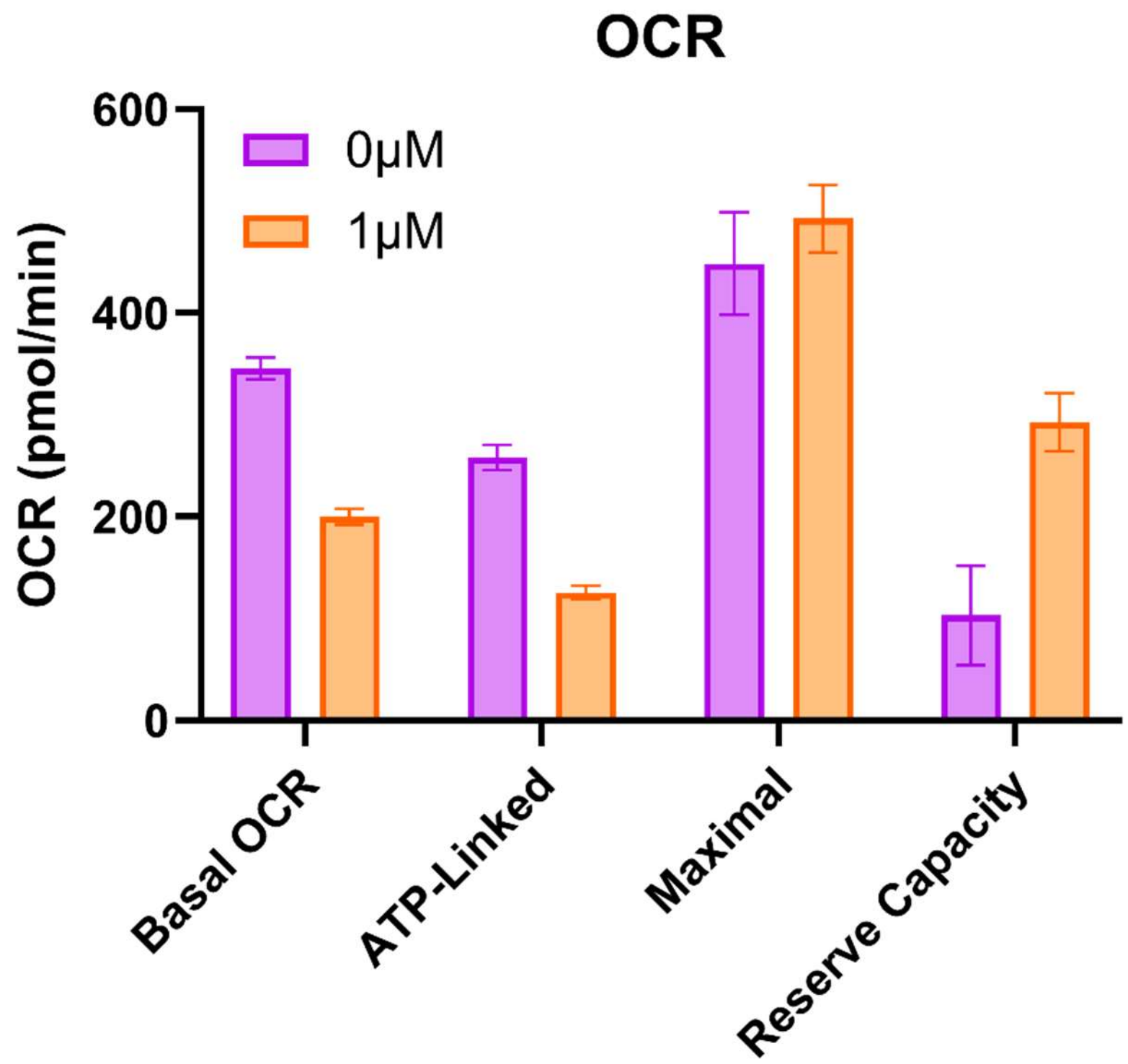

Supplementary Figure S3: Basal, ATP-linked, and maximal oxygen consumption rate (OCR) in addition to the spare reserve capacity at 0μM and 1μM of Navtemadlin (n ≥ 3 biological replicates).

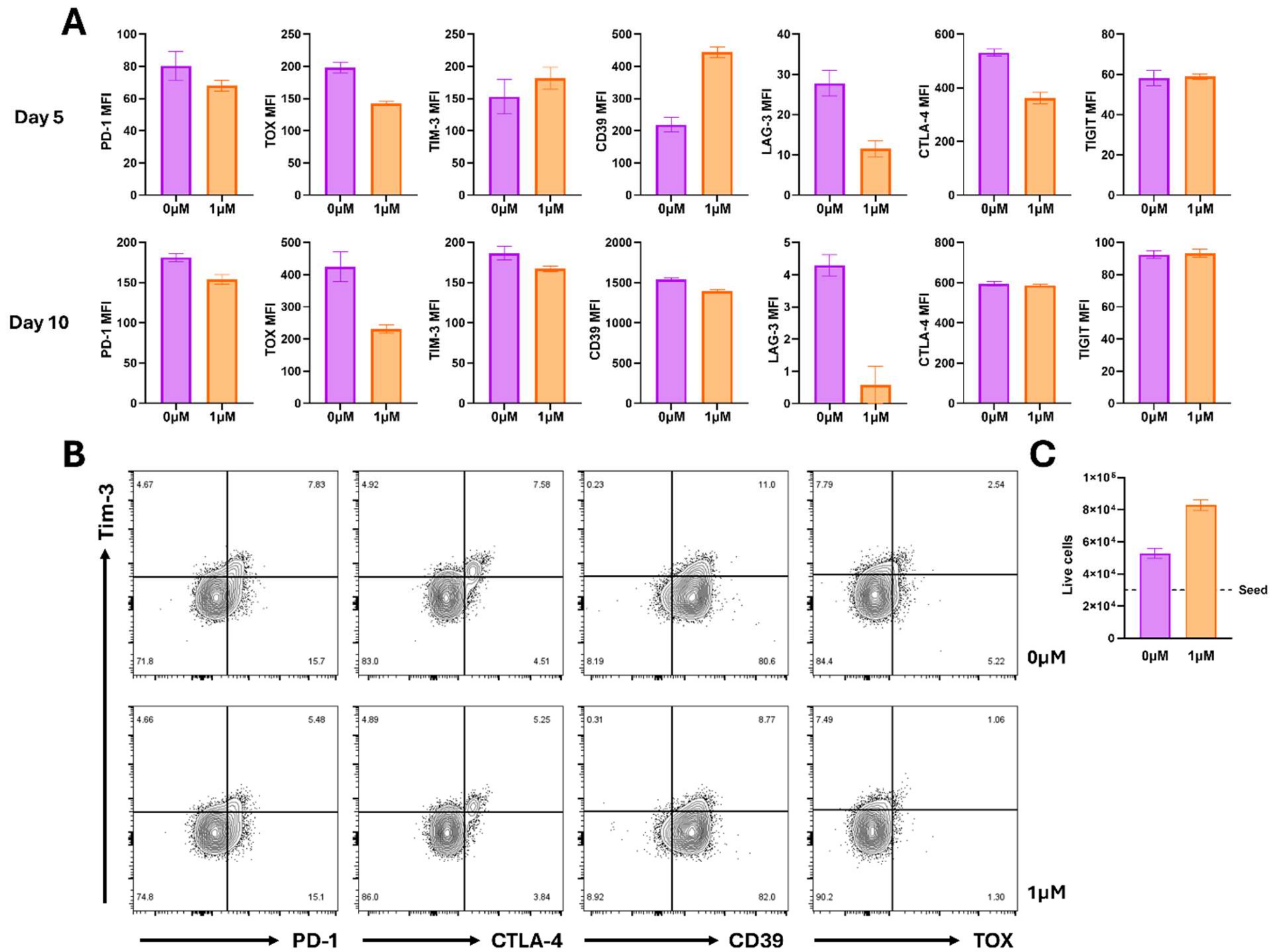

Supplementary Figure S4: Isolated naïve human CD8+ T cells were activated with CD3 and CD28 dynabeads in the presence of IL-2 for 5 days with or without 1μM of Navtemadlin. Fresh media was added on day 2 to mimic TCR transduction step. On day 6, cells from each condition were then transferred into fresh media containing new CD3/CD28 dynabeads but no added IL-2 and cultured for an additional 5 days. All steps and cell numbers used for the manufacturing of TCR-engineered T cells and the tumor co-culture were kept identical. (A) MFI values of ehaustion-associated markers were quantified on day 6 before T-cell restimulation and on day 10. (B) Representative flow plots showed T-cell expression of different exhaustion markers on day 10 when gated on live CD3+ and CD8+. (C) Number of live cells between Navtemadlin-treated and untreated conditions when seeded at the same number of cells on day 6 (n = 4 technical replicates).
